# Treatment of risdiplam after nusinersen continuously improves upper limb motor function in spinal muscular atrophy patients: a multicenter experience

**DOI:** 10.3389/fped.2026.1679549

**Published:** 2026-01-26

**Authors:** Xi Cheng, Yun Ma, Li-Qiang Yu, Ya-Bei Fan, Liang-Hua Zhu, Han-Bing Lu, Qi Niu

**Affiliations:** 1 Department of Geriatrics, The First Affiliated Hospital with Nanjing Medical University, Nanjing Medical University, Nanjing, Jiangsu, China; 2 Department of Neurology, Jiangsu Province (Suqian) Hospital, Suqian, Jiangsu, China; 3 Department of Neurology, The First Affiliated Hospital of Soochow University, Soochow University, Suzhou, Jiangsu, China; 4 Center of Rehabilitation Medicine, The First Affiliated Hospital with Nanjing Medical University, Nanjing Medical University, Nanjing, Jiangsu, China; 5 Department of Pediatrics, The First Affiliated Hospital of Nanjing Medical University, Nanjing Medical University, Nanjing, Jiangsu, China; 6 Department of Neurology, Xuzhou Central Hospital, Xuzhou, Jiangsu, China; 7 Department of Rare Diseases, The First Affiliated Hospital with Nanjing Medical University, Nanjing Medical University, Nanjing, Jiangsu, China

**Keywords:** motor function, nusinersen, real-world data, risdiplam, spinal muscular atrophy

## Abstract

**Introduction:**

Some individuals with spinal muscular atrophy (SMA) transition from nusinersen to risdiplam during disease-modifying therapy (DMT) due to factors such as treatment convenience, economic considerations, and adverse events (AEs). This study evaluates the safety and effectiveness of switching DMTs by analyzing real-world clinical data from multiple centers in China.

**Methods:**

Patients with 5q-SMA who switched from nusinersen to risdiplam were enrolled from four medical institutions in Jiangsu Province. The reasons for switch, as well as any adverse events experienced, were documented. Assessments of motor function were conducted prior to treatment, following the switch, and at four-month intervals subsequently.

**Results:**

A total of eleven patients were included in this retrospective analysis. RULM scores showed maintains improvement following the switch compared to baseline measurements prior to treatment initiation. No significant adverse events were reported after the switch.

**Conclusion:**

Despite the small sample size and lack of a control group, these findings suggest that switching from nusinersen to risdiplam in real-world clinical settings is safe and allows for continued improvement of motor function in SMA patients.

## 1 Introduction

Spinal muscular atrophy (SMA) is a severe and ultimately fatal autosomal recessive disorder caused by mutations in the survival motor neuron 1 (*SMN1*) gene located on chromosome 5q13.2 (1, 2). To date, two disease-modifying therapy (DMT) drugs, Nusinersen and Risdiplam, have been approved in China for the treatment of patients with SMA (3, 4). Nusinersen is an intrathecal antisense oligonucleotide drug targeting the pre-mRNA splicing of *SMN2* gene (5). Previous clinical trials have demonstrated that nusinersen lead to significant enhancements in motor function among SMA patients (1, 3). Risdiplam, an orally administered small molecule, is tailored to selectively modulate the splicing of SMN2 pre-mRNA, facilitating the incorporation of exon 7 and consequently augmenting the levels of functional SMN protein (5). The efficacy of risdiplam has also been established in patients with SMA (6–8).

Given that both drugs target the pre-mRNA of *SMN2*, clinical practice encounters difficulties in determining the appropriate selection between the two medications as well as in managing the transition from one drug to the other. In real-world scenarios, the decision-making process regarding selection of different medications is influenced by economic factors, treatment modalities(intrathecal injection vs. oral route), sociological variables and adverse events (AEs) (9). Previous case series examining switch from nusinersen to risdiplam demonstrated that while both treatments are effective in alleviating symptoms and carry a risk of adverse effects, the majority of participants expressed a preference for the oral administration of risdiplam over the intrathecal delivery of nusinersen (10).Patients, particularly those with severe scoliosis or who have undergone scoliosis treatment, may consider switching from nusinersen to risdiplam for a range of reasons (11). The JEWELFISH study provides some data on exploring the sequential administration of DMT drugs (switch from nusinersen to risdiplam) in individuals with SMA. An increase in serum levels of SMN protein was observed following the administration of risdiplam, with this elevation being sustained over a 24-month treatment period, independent of previous treatments. Exploratory efficacy assessments related to motor function revealed a general stabilization in mean total scores, as measured by the 32-item Motor Function Measure, Hammersmith Functional Motor Scale-Expanded (HFMSE), and Revised Upper Limb Module (RULM) (5). However, real-world evidence regarding switch therapy remains scarce (12). Furthermore, it has been noted that patients may shift from nusinersen to risdiplam without a formal washout period (13). This study aims to evaluate the short-term safety and motor outcome of risdiplam following prior nursinersen therapy by examining its practical implementation in real-world settings in China.

## 2 Material and methods

### 2.1 Patients and procedures

Patients were retrospectively recruited from four medical centers in Jiangsu Province in China, including Jiangsu Province Hospital, Xuzhou Central Hospital, Jiangsu Province (Suqian) Hospital, and the First Affiliated Hospital of Soochow University. This study received approval from the Ethical Review Board of the First Affiliated Hospital with Nanjing Medical University, as well as confirmation from the ethics committee at each participating site. This retrospective study utilized anonymized existing data and posed no risk to participants. Given the retrospective nature and design of the study, the data utilized were anonymized and had been previously recorded, necessitating the implementation of a waiver of informed consent.

Inclusion criteria: (i) confirmation of 5q-SMA according to established diagnostic criteria (14); (ii) prior treatment with nusinersen before switching to risdiplam, no exact duration of prior nusinersen therapy and interval between the last dose and risdiplam initiation; (iii) availability of all prespecified study data and parameters.Exclusion criterion:History of onasemnogene abeparvovec administration. Eligible patients with a confirmed diagnosis of 5q-autosomal recessive SMA switched from nusinersen to risdiplam. Reasons for the switch are detailed in Supplementary Table S1 of the Supplementary Material. Prior to the switch, all patients were intrathecally administered of a 12 mg dose of nusinersen on day 1 (baseline), day 14, day 28, and day 63, followed by maintenance injections every 4 months. Risdiplam was administered orally once daily, with dosages determined by age and body weight: 0.2 mg/kg for infants aged 6 months to less than 2 years, 0.25 mg/kg for patients older than 2 years weighing less than 20 kg, and a fixed dose of 5 mg for patients weighing 20 kg or more. In the following expression, “baseline” refers to the first nusinersen dose; “switch time” refers to the time of conversion to risdiplam.

### 2.2 Motor function

The assessment of motor function was conducted using the HFMSE and the RULM. The HFMSE comprises 33 test items, with higher scores indicating superior motor function. The total score has a maximum of 66 points, and a change of at least 3 points is considered to be clinically significant. The RULM consists of 20 items, with a scoring range from 0 to 37 points. Elevated scores on the RULM are associated with improved arm function, and a minimum increase of 2 points is deemed clinically relevant (1, 15). The 6-minute walk test (6MWT) measured the distance walked by the patient during a 6-minute period.

Patients' independence in daily activities was evaluated by caregivers using the SMA Independence Scale–Upper Limb Module (SMAIS–ULM) (16, 17). Its 22 items primarily evaluate the degree of assistance required for daily tasks.

### 2.3 Statistics analysis

Statistical analyses were performed using SPSS software (version 26.0, IBM Corporation, Armonk, NY, USA). A two-sided *p*-value of less than 0.05 was considered to indicate statistical significance. The Wilcoxon matched-pairs, signed-rank test was employed to assess alterations in motor function outcomes from baseline to the time of switch and thereafter.

## 3 Results

### 3.1 Demographic and clinical characteristics

**Figure 1:**
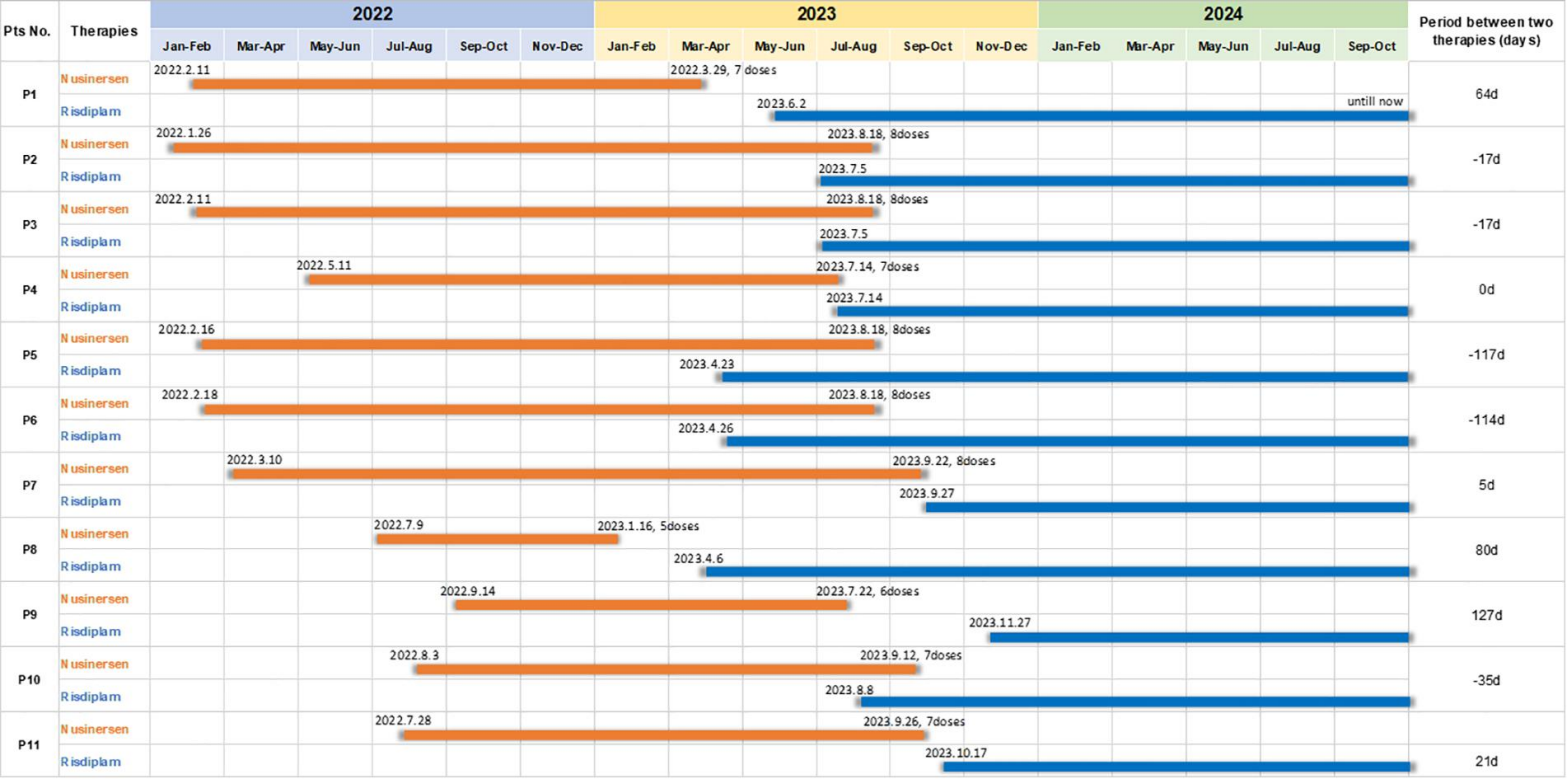
The timeline nfor transitioning from nusinersen to risdiplam.

The study involved a cohort of 11 patients diagnosed with SMA from four distinct medical centers who underwent a switch therapy from nusinersen to risdiplam. The clinical characteristics and demographic information are presented in Table 1. The patient population included individuals with SMA type 1, 2, and 3, with type 3 constituting 27.3%, type 2 63.6%, and type 1 9% of the total sample. The age at which nusinersen was initiated ranged from 5 to 11 years, with a median age of 9 years. The number of doses of nusinersen administered varied from 5 to 8 times, with a median of 7 doses. The initiation age for risdiplam ranged from 6 to 12 years, with a median age of 11 years. The timeline for the switch from nusinersen to risdiplam is illustrated in Figure 1. During the transition, five patients experienced a period of combination of nusinersen and risdiplam. Only one patient (P9) had received the last dose of nusinersen more than 90 days before initiating risdiplam treatment. The median follow-up duration in our study is 8 months.

**Table 1:** Clinical characterization of patients with SMA.

Patient	Gender	SMA subtype	Functional status	Age at first dose nusinersen, years	Number of doses of nusinersen	Age at first dose risdiplam, years
P1	Male	3	walk	10	7	11
P2	Male	2	sit	9	8	11
P3	Female	2	sit	9	8	10
P4	Male	3	walk	11	7	12
P5	Male	2	sit	8	8	9
P6	Male	3	sit	9	8	11
P7	Female	2	un-sit	10	8	11
P8	Female	2	un-sit	5	5	6
P9	Female	2	sit	9	6	10
P10	Male	2	un-sit	11	7	12
P11	Male	1	un-sit	9	7	10

### 3.2 Motor function outcomes

**Figure 2:**
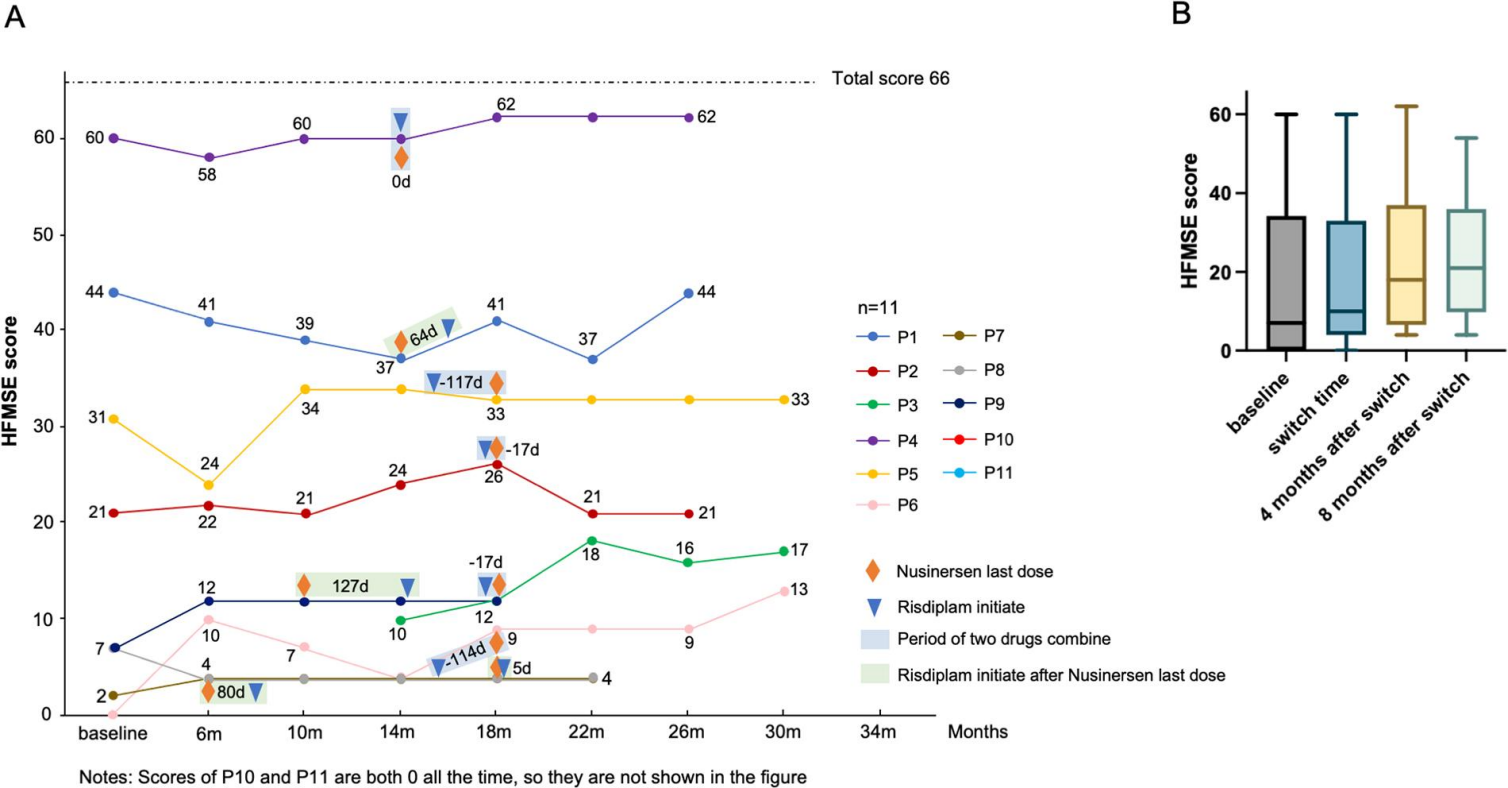
The HFMSE scores at baseline, at the switch time, and throughout the follow-up. **(A)** The motor function scores of eleven SMA patients on the HFMSE scale. **(B)** Comparison of HFMSE scores at different time points.

The motor function scores of eleven SMA patients on the HFMSE scale are presented in Figure 2A, including baseline measurements (17.2 ± 21.3), the time of switch (17.7 ± 19.1), and subsequent follow-up assessments (four months after switch: 18.6 ± 19.6; eight months after switch: 19.1 ± 17.8). Although these changes did not reach statistical significance, a consistent upward trend in HFMSE scores was observed following the switch and throughout the observation period (Figures 2A,B and Supplementary Table S2). Compared with the time of switch, two patients demonstrated clinically significant improvements in HFMSE scores (defined as an improvement of ≥3 points) after four months of follow-up, with one patient exhibiting similar enhancement at the eight-month follow-up. One patient exhibited a clinically significant decline in HFMSE scores (defined as an worsing of ≥3) at four months, and two patients showed worsening at eight months compared to the time of switch (Table 2).

**Table 2:** Changes from baseline to 8 months after switch in motor function assessments.

Motor function assessments	Switch time vs. baseline	4 months after switch vs. baseline	4 months after switch vs. switch time	8 months after switch vs. baseline	8 months after switch vs. switch time
HFMSE++	3/10	2/10	2/11	2/10	1/10
HFMSE–	2/10	2/10	1/11	3/10	2/10
RULM++	5/10	7/9	2/9	5/9	1/10
RULM–	0	0	2/9	0	0
6MWT (meters)	−11	+69	+80	+39	+50

HFMSE++ HFMSE improvement ≥3.

HFMSE– HFMSE worsening ≥3.

RULM++ RULM improvement ≥2.

RULM– RULM worsening ≥2.

6MWT 6-minute walk test.

vs. versus.

**Figure 3:**
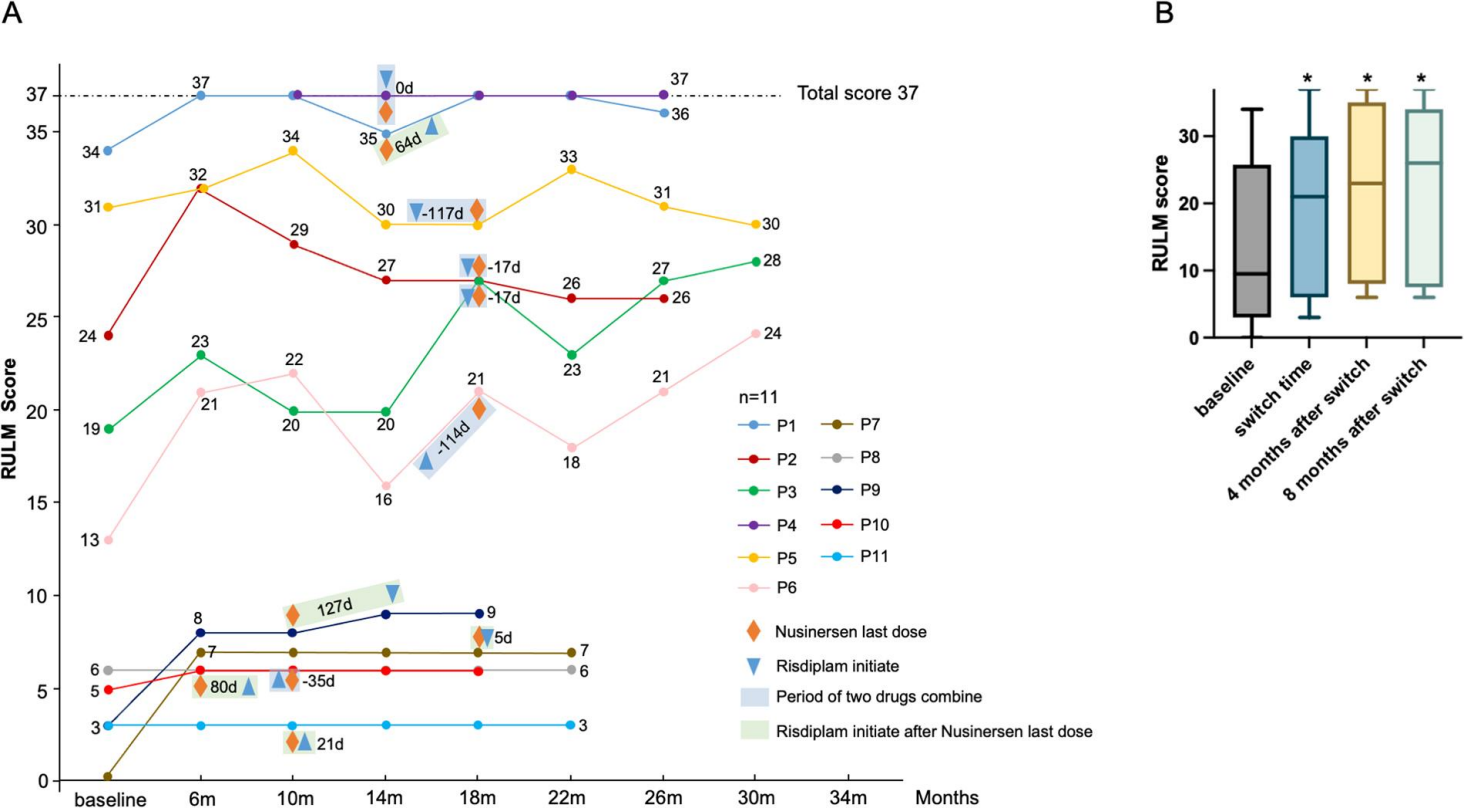
The RULM scores at baseline, at the switch time, and throughout the follow-up. **(A)** The motor function scores of eleven SMA patients on the RULM scale. **(B)** Comparison of RULM scores at different time points. * *P* < 0.05 compared with baseline.

The motor function scores of eleven SMA patients on the RULM scale are showen in Figure 3A. An upward trend in RULM median scores was observed following the switch and throughout the observation period (Supplementary Table S2). The RULM score at the time of switch (18.82 ± 13.0) was significantly higher than the baseline score (13.8 ± 12.5) (*P* = 0.024). A significant increase in RULM scores was also observed four months after the switch (21.8 ± 12.5) compared to baseline (*P* = 0.018) and this improvement remained statistically significant eight months post-switch (20.3 ± 13.3, *P* = 0.027). However, no significant differences were found in RULM scores four or eight months after the switch when compared to the time of switch (Figure 3B). Specifically, seven patients achieved clinically meaningful improvements in upper limb function four months after the switch, while five patients exhibited similar enhancements at the eight-month follow-up. Additionally, two patients showed further clinically significant improvements in upper limb function four months after the transition, and one patient exhibited a notable enhancement at the eight-month follow-up. Conversely, two patients experienced a decline in upper limb function scores four months following the switch, whereas no patients showed a significant decrease in upper limb function at the eight-month follow-up. (Table 2).

The 6MWT was performed in one ambulatory patient with SMA type 3 (Patient 4). Compared with baseline 314 meters(m), the walking distance decreased at switch time (–11 m), improved at four months post-switch (+69 m), and remained increased at eight months post-switch (+39 m). Relative to switch time 303 m, the walking distance increased by 80 meters at four months post-switch and by 50 meters at eight months post-switch (Table 2). In the only patient who completed the 6MWT assessment, we observed a positive trend. Given that this is only a single case, we consider it an anecdote.

### 3.3 Patient independence

The SMAIS–ULM scores were recorded both before and after the switch. It is noteworthy that, since the assessment was conducted after the commencement of the study, a baseline assessment was not available for certain participants. SMAIS–ULM scores data were obtained for 3 patients prior to the switch, with a median score of 27 (range 20–41).The SMAIS–ULM scores of 9 patients were obtained 8–12 months post-switch, with a median score of 21 (range 8–39). In the bathing/personal hygiene domain, 2 patients were unable to complete the tasks without full assistance, while the remaining patients required partial assistance. In the dressing domain, 6 patients were unable to dress independently and required full assistance, whereas 1 patient could complete the task unaided. In the eating domain, 1 patient required complete assistance, while the others needed partial help.

### 3.4 Safety outcomes

Adverse event collection was primarily based on medical record reviews. By September 4, 2024, no serious adverse events had been reported in our study. By the end of the follow-up period, only case 9, a patient diagnosed with SMA type 2, exhibited symptoms of weight loss, diminished appetite, and hair loss, however, the patient remained compliant with their prescribed medication regimen. Compared with the spectrun of spontaneous adverse drug reactions (ADRs) associated with DMTs for SMA reported in a recent comprehensive post-marketing safety analysis (18), weight loss, diminished appetite, and hair loss may represent potential novel ADRs.

## 4 Discussion

This retrospective, multicenter study evaluated the real-world transition from nusinersen to risdiplam in patients with SMA over a maximum follow-up period of 12 months. a maximum follow-up period of 12 months.

We summarized the reasons for switching between the two therapies. The primary reason identified in our study was the difficulty associated with intrathecal injections, particularly in patients with severe scoliosis or those who had undergone spinal fusion surgery findings consistent with those reported in similar conversion studies (11, 19). Additionally, five patients opts to switch to other medications due to concerns regarding the invasive nature of lumbar punctures. Nusinersen was first DMT for SMA included in China's medical insurance catalog, followed later by the risdiplam. In certain urban regions, the cost of risdiplam is lower than that of nusinersen for specific patients due to the advantages of health insurance. Concequently, three patients switched treatments due to financial constrains. Furthermore, one patient switched to risdiplam following the onset of post-lumbar puncture headaches subsequent to nusinersen administration. From the standpoint of convenience alone, oral risdiplam is clearly more favorable than intrathecal nusinersen. However, clinical decision-making should also consider therapeutic efficacy, safety profile, cost-effectiveness, and individual patient circumstances.

In the JEWELFISH study (5), a three-month washout period following nusinersen administration was considered necessary before initiating risdiplam, in order to assess the tolerability and pharmacokinetic profile of risdiplam after switching from nusinersen. In the study by Yan et al. (13), eight pediatric patients initiated risdiplam within 30 days after their last nusinersen dose, two patients received treatment between 31 and 60 days post-nusinersen, and five patients started risdiplam between 61 and 89 days after the final nusinersen dose. Safety data from patients who initiated risdiplam within a 90-day washout period following nusinersen showed favorable outcomes. Furthermore, the majority of patients in our study did not observe a 90-day washout period. The median transition interval in our cohort was 0 days, with a range from −117 to 127 days. Despite the non-standardized washout period, patients in our study demonstrated both safety and efficacy.

We synthesized existing data on therapy switching from nusinersen to risdiplam in SMA (Supplementary Table S3). Current conversion studies primarily involve patients with SMA types 1, 2, and 3, with heterogeneous follow-up durations. Assessments of motor function remain inconsistent across studies. In our study, we observed a significant increase in RULM scores after initiation of DMT compared to baseline levels, with scores remaining stable after the treatment switch. It is speculated that the majority of patients in our study were categorized as type 2 and type 3 categories, with relatively well-preserved upper limb motor function. Our findings indicate a statistically significant enhancement in upper limb motor function relative to pre-treatment levels. It was onsistent with the findings from JEWELFISH study, RULM scores showed a sustained upward trend at both 12 and 24 months post-switch, with an average increase of 0.16 recorded at the 12-month mark (5). Although no statistically significant difference was observed when comparing post-switch assessments to the time of switch, an increasing trend in both HFMSE and RULM scores was evident. Ebru Bekircan-Kurt et al. (20) examined motor outcomes following the switch from nusinersen to risdiplam in a single-center study and reported that motor function improved following the initiation of nusinersen treatment, with the most substantial gains observed during the first year. After switching to risdiplam, motor function was largely maintained findings that align with the those of our study.

Among ambulatory patients, the JEWELFISH study (5) reported a mean increase of 30.88 meters in total walking distance at of 24-month post-switch compared to baseline in eight patients. Similarly, the 6MWT results showed clear improvement at 4 and 8 months post-switch relative to pre-switch values in our study. But only one ambulatory patient was included in our cohort, it is essentially anecdotal and must be interpreted with great caution. These results further support the presercation of ambulatory capacity following the switch in treatment. Additionly, our study included a notable type 1 SMA patient(case11), who was unable to sit independently prior to DMT initiation. Following the switch to risdiplam,his RULM score remained stable. This observation is consistent with finding from Ribeiro et al., which suggest that risdiplam may improve survival and motor function in type 1 SMA patients compared to nusinersen (21).

Since the introduction of DMTs for SMA into clinical practice, comprehensive evaluation of their safety profiles have conducted. Approximately 16% of participants treated with risdiplam reported adverse events with pharmacovigilance data indicating a predominant occurrence of gastrointestinal and respiratory ADRs (18, 22, 23).

In the nusinersen switch to risdiplam therapy subgroup of the JEWELFISH study (5), 96% of patients experienced treatment-related AEs, including fever, upper respiratory tract infections, headache, nasopharyngitis, diarrhea, nausea and cough. Notably, a one-year observational study on switching from nusinersen to risdiplam in Croatian reported no cases of respiratory or feeding deterioration, nor any fatal events (24). A review on DMTs in SMA patients concluded that switching from nusinersen to risdiplam considered safe based on emerging clinical and real-world evidence (12).Among the subjects analyzed in our study, only one case, a patient diagnosed with SMA type 2, exhibited weight loss, decreased appetite, and alopecia. This low incidence may be attributed to underreporting,as some adverse events occurring during the study period might not have been fully documented in medical records (Supplementary Table S3).

In addition, the JEWELFISH study showed an increase in total SMAIS-ULM scores as reported by caregivers over a 24-month duration, with a mean increase of 1.90 from baseline (5). In our study, there was no noted increase in the SMAIS-ULM score following the switch. The scarcity of data, with only three cases available for pre- and post-analysis, may be a relevant consideration.

Our study is an exploratory, observational case series and has several limitations. First, our study is limited by a small number of cases involving treatment switching, and subgroup analyses according to disease duration were not conducted. Caution should be exercised when interpreting the data from this heterogeneous cohort, given the variation in disease duration. To substantiate our findings, larger-scale studies are warranted. Second, this was a retrospective study, cliniacal data such as the documentation of adverse events was incomplete. In the original study design, the primary endpoint was motor function score, and a standardized quality of life assessment tool was not systematically incorporated. The SMAIS–ULM scores lacked data for comparison baseline and follow-up assessments in this study.Third, the maximum follow-up duration was 12 months, which may be insufficient to fully evaluate long-term prognosis; a longer observation period is needed. Fourth, the absence of a control group limits comparability, as this is a rare disease cohort with few untreated patients. Fifth, due to the multicenter design, there may be variability in motor function assessments across sites. To minimize this potential bias, each patient's pre- and post-treatment evaluations were conducted by the same assessor. Despite these limitations, our results suggest that switching from nusinersen to risdiplam at any disease stage is both safe and effective in patients with spinal muscular atrophy (SMA), and may support clinicians in optimizing patient management.

## 5 Conclusions

This multicenter study evaluated the real-world transition from nusinersen to risdiplam in patients with SMA across four centers. No significant adverse events were observed following the switch. Motor function, particularly upper limb function, either remains stable or improve after treatment conversion.The results suggest that switching from nusinersen to risdiplam in clinical practice is safe and associated with sustained or improved motor outcomes in patients with SMA.

## Data Availability

The raw data supporting the conclusions of this article will be made available by the authors, without undue reservation.
