# Supplementary material for: Treatment of risdiplam after nusinersen continuously improves upper limb motor function in spinal muscular atrophy patients: a multicenter experience
Source: Front Pediatr. 2026 Jan 26;14:1679549. doi: 10.3389/fped.2026.1679549 (PMC12883809; doi:10.3389/fped.2026.1679549)
Supplement: Supplementary file 1 [file Table1.docx]

Supplementary Table 1. Reported reasons why patients began treatment with risdiplam following nusinersen

| Reasons to switch from nusinersen to risdiplam | n = 11 |
| --- | --- |
| Difficulty to receive a lumbar puncture ^a^ | 9 (82%) |
| Treatment response: lack of efficacy | 1(9%) |
| Fear of pain from lumbar puncture | 5 (45%) |
| Economic reasons ^b^ | 3(27%) |
| Adverse reaction after nusinersen treatment | 1(9%) |

^a^ Difficulty to receive a lumbar puncture refers to challenges in administering intrathecal treatments to individuals with scoliosis or previous spinal surgery.

^b^ Economic reasons refer to risdiplam costs less than nusinersen with the help of health insurance
