# Supplementary material for: Treatment of risdiplam after nusinersen continuously improves upper limb motor function in spinal muscular atrophy patients: a multicenter experience
Source: Front Pediatr. 2026 Jan 26;14:1679549. doi: 10.3389/fped.2026.1679549 (PMC12883809; doi:10.3389/fped.2026.1679549)
Supplement: Supplementary file 2 [file Table2.docx]

Supplementary Table **2** Motor Function Assessments from baseline to 8 months after switch

|  | baseline | switch time | 4 months after switch | 8 months after switch |
| --- | --- | --- | --- | --- |
| HFMSE（med） | 7 | 10 | 18 | 21 |
| HFMSE（IQR） | 28 | 25.5 | 24 | 22.75 |
| RULM (med) | 9.5 | 21 | 23 | 26 |
| RULM (IQR) | 19.25 | 22 | 24 | 22 |

med: median

**IQR: Interquartile Range**
