# Supplementary material for: Treatment of risdiplam after nusinersen continuously improves upper limb motor function in spinal muscular atrophy patients: a multicenter experience
Source: Front Pediatr. 2026 Jan 26;14:1679549. doi: 10.3389/fped.2026.1679549 (PMC12883809; doi:10.3389/fped.2026.1679549)
Supplement: Supplementary file 3 [file Table3.docx]

**Supplementary Table 3** Clinical Researches on Nusinersen Switch to Risdiplam

| **First author** | **population（n）** | **SMA type** | **Follow-up time after conversion** | **main outcomes** | **mentioned adverse drug reactions (ADRs)** | **Incidence of ADRs** |
| --- | --- | --- | --- | --- | --- | --- |
| Chiriboga CA[5] | 76 | 1(12%)  2 (57%)  3 (32%) | 2years | HFMSE,MFM32,RULM,6MWT | Pyrexia,URTI,Headache, Nasopharyngitis,Diarrhea,Nausea,Cough,Pneumonia,Respiratory failure, Respiratory distress, LRTI | 96% |
| Can Ebru Bekircan-Kurt[19] | 44 | 1(25%)  2(56.8%)  3(18.2%) | 2years | CHOP-INTEND,HFMSE,RULM,Pulmonary fucntion,Feeding,Hospitalization | diarrhea and mild gastrointestinal symptoms，transient tachycardia and rash | 9% |
| Andrej Belancˇic ́ [21] | 17 | 1（35.3%）  3（64.7%） | 12 months | CHOP-INTEND,HFMSE,RHS,Respiratory support,feeding support | weight gain | / |
| Our study | 11 | 1(9%)  2(63.6%)  3(27.3%) | 12 months | HFMSE,RULM, 6MWT | weight loss, diminished appetite, and hair loss | 9% |
